# Is generalization of exhaled CO assessment in primary care helpful for early diagnosis of COPD?

**DOI:** 10.1186/s12890-015-0039-6

**Published:** 2015-04-28

**Authors:** Nicolas Molinari, Mathieu Abou-Badra, Grégory Marin, Chin-Long Ky, Noemi Amador, Anne Sophie Gamez, Isabelle Vachier, Arnaud Bourdin

**Affiliations:** Département de l’Information Médicale. Hôpital La Colombière, CHU Montpellier, Montpellier, France; PhyMedExp, University of Montpellier, INSERM U1046, CNRS UMR 9214, Montpellier, France; Deptartment of Respiratory Disease, CHU Montpellier, Montpellier, France; Ifremer, UMR EIO241, CIP, BP 7004, 98719 TARAVAO, Tahiti Polynésie Française

**Keywords:** COPD, Screening, Exhaled CO, Smoking, Behaviour

## Abstract

**Background:**

COPD is largely under-diagnosed and once diagnosed usually at a late stage. Early diagnosis is thoroughly recommended but most attempts failed as the disease is marginally known and screening marginally accepted. It is a rare cause of concern in primary care and spirometry is not very common. Exhaled carbon monoxide (eCO) is a 5-seconds easy-to-use device dedicated to monitor cigarette smoke consumption. We aimed to assess whether systematic eCO measurement in primary care is a useful tool to improve acceptance for early COPD diagnosis.

**Methods:**

This was a two-center randomized controlled trial enrolling 410 patients between March and May, 2013. Whatever was the reason of attendance to the clinic, all adults were proposed to measure eCO during randomly chosen days and outcomes were compared between the two different groups of patients (performing and not performing eCO). Primary outcome was the rates of acceptance for COPD screening.

**Results:**

Rate of acceptance for COPD screening was 28% in the eCO group and 26% in the other (P = 0.575). These rates increased to 48 and 51% in smokers (current and former). eCO significantly increased the rate of clinics during which a debate on smoking was initiated (42 vs. 24%, P = 0.001). eCO at 2.5 ppm was the discriminative concentration for identifying active smokers (ROC curve AUC: 0.935). Smoking was the only independent risk factor associated with acceptance for early COPD screening (OR = 364.6 (82.5-901.5) and OR = 78.5 (18.7-330.0) in current and former smokers, respectively) while eCO measurement was not.

**Conclusions:**

Early COPD diagnosis is a minor cause of concern in primary care. Systematic eCO assessment failed to improve acceptance for early COPD screening.

## Background

Increasing concerns about Chronic obstructive pulmonary disease (COPD) are raised since it will be the fourth leading cause of deaths by 2030 [1]. Under diagnosis of COPD is a global problem, delaying adequate treatment and the possibility of preventing physical, emotional and socioeconomic consequences of the disease [2]. Multiple attempts aimed at diagnosing COPD earlier but to date none really penetrated the primary care in western countries. Early detection of COPD is crucial for promoting smoking cessation – which is more or less the unique way to interfere with the natural history of the disease. The under diagnosis of COPD is mostly related to spirometry pitfalls which included- but not restricted to-physicians- and patients-related factors. The reasons for general practitioners (GP) not performing spirometry might be limited access to the equipment, lack of adequate training and time constraints [2]. On the patients ‘side, under diagnosis may be a result of the gradual adaptation to increasing shortness of breath due to declining lung function and a reluctance to seek for medical advice before severe symptoms occur [3]. Moreover minor knowledge of the disease towards the general population leads to a non-discussion about shortness of breath: “Surrounding COPD is an historical nihilism, with patients and even their doctors establishing blame and blatantly denying a medical problem exists” was written in a review in 2009 [2]. Recently, the relative stability of the phenotype toward exacerbations rates identified a subgroup of COPD patients with a very low disease-related risk in terms of hospitalizations, exacerbation, accelerated decline in lung function or comorbidity [4,5]. This fact did not help for persuading GPs that early COPD diagnosis is a worth effort. Questionnaires have been developed and more or less successfully developed but the issue raised by most GPs associations dealt with an unacceptable number of questionnaires that might be applied routinely for most chronic diseases [6,7].

Assessing exhaled carbon monoxide (eCO) concentration is routinely used in tobacco weaning programs for up to fifteen years. It was shown as a valuable noninvasive biomarker of cigarette smoke daily consumption which passed through most validation studies. As it stands, eCO is a 5 or 10 seconds measurement that responds to most issues related to any tool: easy to do, no contraindication, no expertise requirement, absolute harmlessness, low cost [8].

As cigarette smoking remains the main cause of COPD in western countries, we hypothesized that assessing eCO in primary care will help GPs to improve awareness about COPD that will introduce acceptance for a COPD screening. We assumed that eCO assessment in waiting rooms will improve the debate on smoking and COPD during medical consultations.

Accordingly, we aimed to test this hypothesis in primary care through a two-center randomized controlled trial.

## Methods

### Study design and population

A prospective multicenter study was conducted in two outpatient primary care offices. All adult patients attending the outpatient clinic were consecutively included. The study was proposed to any patients who turned up at the clinics during the study period. Exclusion criteria were refusal to participate after information and age under 18 years old. Once attending the GP’s office, the investigator presented the study and gathered the non opposition approval. In the waiting room, a junior doctor (not the GP) fulfilled the Case Report Form (CRF) gathering the following information: age, gender, smoking status (current, former: 6 months of smoking abstinence, never). eCO concentration was assessed in the waiting room on randomly chosen days and other days considered as a control. In this study, we randomized the days and not the subjects. The randomization of days was done once for the study period to obtain balanced samples of patients. All patients were consecutively included with or without eCO assessment depending on the randomization table.

Afterwards, during medical consultations, the junior doctor observed if concerns about smoking were raised and if any awareness about COPD were discussed and who initiated it (the patient or the GP). In the end both doctors were the ones who would consider if the patient declared acceptance for a COPD screening. However, we didn’t know if the patients finally had an appointment with the pulmonologist. Note that the GPs did not modify their usual practice as medical consultations were conducted in a conventional way.

From March 2013 to May 2013 (3 months), 410 consecutive patients were screened and enrolled.

### Ethics and consent

The local ethics committee “Comité de Protection des personnes Sud-Mediterranee III” approved the study design (code UF: 9134, register: 2013-A00104-41). Because of the non invasive design of this study, a non opposition statement was obtained for all included patients.

### Statistical analysis

The primary outcome was the comparison of rates of patient’s acceptance of a COPD screening between the two different groups of patients (performing and not performing eCO). The number of subjects needed was calculated by assuming that this rate will reach 15% when exhaled CO levels were measured and remained equal or below to 5% without exhaled CO measurements. Assuming a two-sided alpha risk of 0.05 and a beta risk of 0.05, we calculated that at least 219 patients per group would be required to identify a difference of 10%.

Outcome variables were acceptance for COPD screening, awareness for COPD and debate on smoking. Predictors were gender, age, smoking status, pack-years and patient’s group (performing or not performing eCO).

Data are expressed as mean ± SD for continuous variables (age, pack-years, exhaled CO), or as number and percentage for categorical variables (gender, tobacco status). Continuous variables were compared using Student’s *t*-test for normally distributed variables or Mann-Whitney rank-sum test for non-normally distributed variables. Pearson’s Chi-squared test or Fisher exact test was used to compare categorical variables. We did not use any penalization for multiple testing procedures. Multivariate models were established. Significance was established at *P*< 0.05. Kernel density estimate was used with a Gaussian kernel to estimate the exhaled CO distribution. The thresholds of CO values to predict smokers and non smokers were assessed using a Receiver Operating Characteristic (ROC) curve analysis. Statistical analysis was performed by an independent statistician, with R software (version 2.15.2).

## Results

Between March and May 2013, 410 patients were included in two different offices. Measurement of exhaled CO was conducted in 216 patients. Patients’ characteristics are presented in Table 1. Fifty-three percent were females and the mean age (± standard deviation) was 61 ± 16 years. Twenty-three and 32% were current and former smokers respectively. Within smokers and former smokers, mean ± standard deviation cigarette smoking history was 22 ± 16 pack-years.

**Table 1:** **Characteristics of the study population**

**Variable**	**All**	**Without eCO assessment**	**With eCO assessment**	***P-value***
	**(n = 410)**	**(n = 194)**	**(n = 216)**	
Female (%)	216 (53.3)	103 (53.9)	113 (52.8)	.875^c^
Age (years)	60.6 ± 16.2	61.8 ± 15.8	59.5 ± 16.5	.144^t^
Smoking status				.095^c^
*Current smokers (%)*	96 (23.4)	37 (19.1)	59 (27.3)	
*Former smokers (%)*	130 (31.7)	61 (31.4)	69 (31.9)	
*Never smokers (%)*	184 (44.9)	96 (49.5)	88 (40.7)	
Pack-years	22.4 ± 15.9	22.4 ± 16.6	22.3 ± 15.6	.918^m^
Exhaled CO (ppm)	3.88 ± 7.01	NA	3.88 ± 7.01	NA

(t) Student t-test, (m) Mann-Whitney rank sum test, (c) Pearson’s Chi-squared test.

Categorical data are defined in number and percentage. Continuous data are expressed as mean and standard deviation. Comparisons were made between patients who ended up with an exhaled CO measurement and patients without measurement.

**Figure 1:**
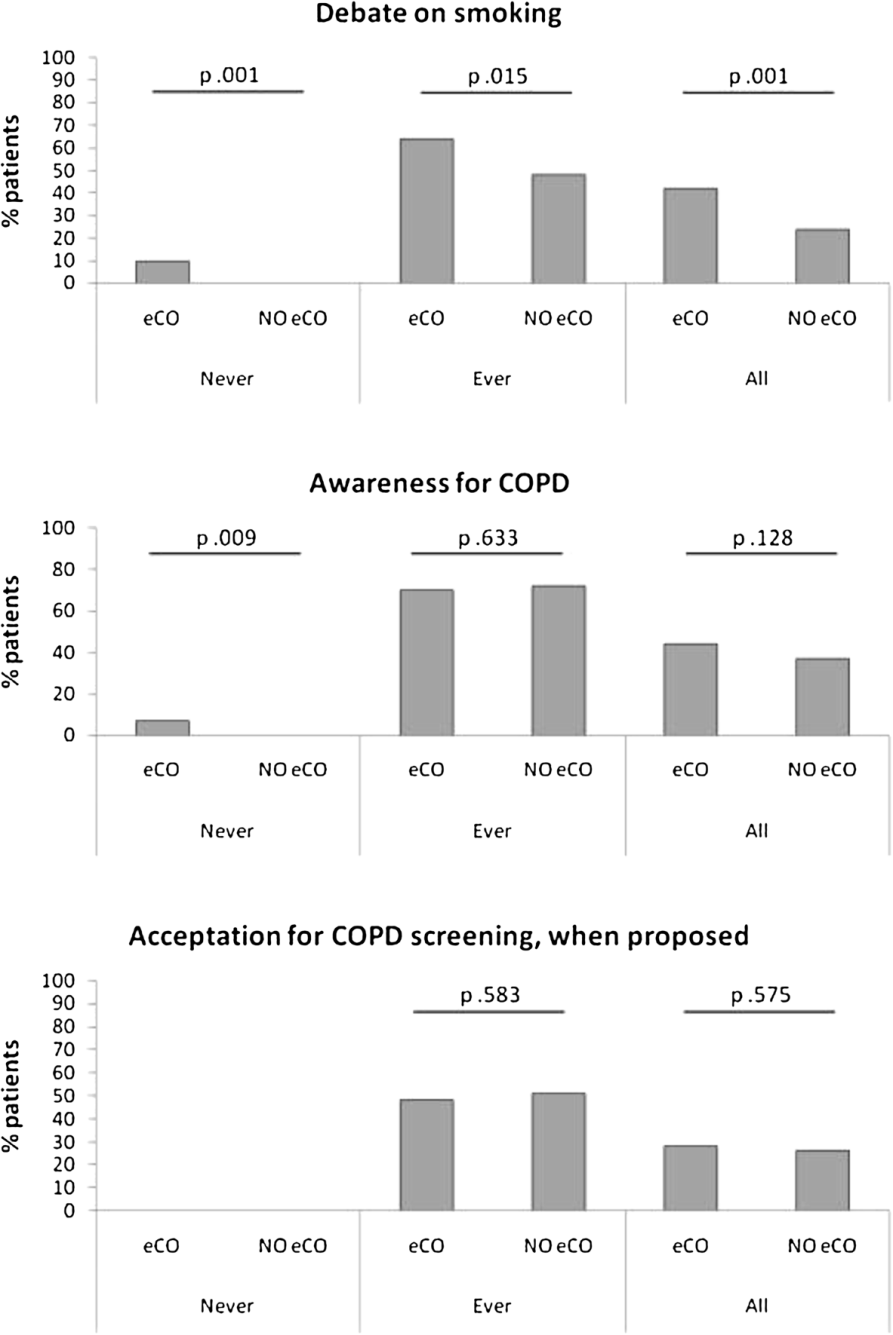
Outcomes according to patient’s groups and smoking status.

Cigarette smoking was discussed with up to 138 different patients (33.7%). Table 2 presents the outcomes according to patient groups and smoking status. From these 138 consultations, 91 have been subjected to CO measurements (P < 0.001). Forty-three out of 138 consultations, the debate was initiated by the patient himself while 95 were introduced by the physician. Taking into account the 166 debates in which awareness about COPD was discussed, 95 had been subjected to CO measurements (P = 0.128). COPD debate was mostly introduced by the physician (149 out of 166). In the end, 111 patients agreed on entering a COPD screening process, 61 being subjected to CO measurements (P = 0.576). Figure 1 displays differences in outcomes according to patient’s groups and smoking status.

**Table 2:** **Outcomes according to patient groups and smoking status**

**Outcomes**	**eCO performing**			**No eCO performing**			**Total**			***p*** **-value**		
										**(eCO vs No eCO)**		
**Smoking subgroup**	**Never (n = 88)**	**Ever (n = 128)**	**All (n = 216)**	**Never (n = 96)**	**Ever (n = 98)**	**All (n = 194)**	**Never (n = 184)**	**Ever (n = 226)**	**All (n = 410)**	**Never (n = 184)**	**Ever (n = 226)**	**All (n = 410)**
**Debate on smoking, n(%)**	**9 (10)**	**82 (64)**	**91 (42)**	**0 (0)**	**47 (48)**	**47 (24)**	**9 (5)**	**129 (57)**	**138 (34)**	**0.001** ^**f**^	**0.015**	**<0.001**
Initiated by GP	2	53	55	0	40	40	2	93	95	NA	0.923	0.246
Initiated by patient	7	29	36	0	7	7	7	36	43	NA	0.002	<0.001
**Awareness for COPD, n(%)**	**6 (7)**	**89 (70)**	**95 (44)**	**0 (0)**	**71 (72)**	**71 (37)**	**6 (3)**	**160 (71)**	**166 (40)**	**0.011** ^**f**^	**0.633**	**0.128**
Initiated by GP	4	78	82	0	67	67	4	145	149	NA	0.248	0.471
Initiated by patient	2	11	13	0	4	4	2	15	17	NA	0.177	0.045
**Acceptance for COPD screening, when proposed, n(%)**	**0 (0)**	**61 (48)**	**61 (28)**	**0 (0)**	**50 (51)**	**50 (26)**	**2 (1)**	**111 (49)**	**111 (27)**	**NA**	**0.616**	**0.575**

Comparisons are done with Pearson’s Chi-squared test or Fisher exact test (f).

Univariate and multivariate logistic regressions for the three outcomes are presented in Table 3. Smoking status and eCO measurement were independent factors associated with the occurrence of a debate on smoking during the medical consultation with odds ratios of 34.8 (95% CI = 11.0 to 133, *P* < 0.001) in current smokers and 11.8 (95% CI = 5.3 to 26.2, *P* < 0.001) in former smokers, and 2.56 (95% CI = 1.45 to 4.51, *P* = 0.001) for eCO assessment.

**Table 3:** **Univariate and multivariate analyses for each of the tree outcomes**

**Variable**	**Univariate**		**Multivariate**	
	**Odds-ratio**	***p-value***	**Odds-ratio**	***p-value***
**Outcome 1: debate on smoking**				
Female (%)	0.59 (0.39 - 0.91)	0.015	0.85 (0.48 - 1.53)	0.596
Age (years)	0.96 (0.94 - 0.97)	<0.001	0.98 (0.96 - 1.00)	0.104
Tobacco		<0.001		<0.001
*Current smokers*	90.4 (38.6 - 211.5)	<0.001	38.3 (11.0 - 133.1)	<0.001
*Former smokers*	12.1 (5.69 - 25.9)	<0.001	11.8 (5.32 - 26.2)	<0.001
*Never smokers*	1		1	
Pack-years	1.01 (0.97 - 1.05)	0.553	1.02 (0.96 - 1.05)	0.844
Exhaled CO performing	2.24 (1.47 - 3.43)	0.001	2.56 (1.45 - 4.51)	0.001
**Outcome 2: awareness for COPD**				
Female (%)	0.46 (0.31 - 0.69)	0.001	0.76 (0.41 - 1.39)	0.369
Age (years)	0.96 (0.95 - 0.97)	<0.001	0.99 (0.97 - 1.02)	0.676
Tobacco		<0.001		<0.001
*Current smokers*	255.1 (89.8 - 724.9)	<0.001	48.01 (10.7 - 215.0)	<0.001
*Former smokers*	39.2 (16.2 - 94.9)	<0.001	35.8 (14.4 - 88.7)	<0.001
*Never smokers*	1		1	
Pack-years	1.08 (1.01 - 1.17)	0.032	1.03 (0.96 - 1.17)	0.082
Exhaled CO performing	1.34 (0.90 - 1.99)	0.150	0.98 (0.55 - 1.76)	0.959
**Outcome 3: acceptance for COPD screening**				
Female (%)	0.59 (0.39 - 0.88)	0.011	1.21 (0.65 - 2.12)	0.548
Age (years)	0.96 (0.94 - 0.97)	<0.001	0.97 (0.95 - 1.02)	0.127
Tobacco		<0.001		<0.001
*Current smokers*	394.3 (89.3 - 896.4)	<0.001	73.67 (12.9 - 423.1)	<0.001
*Former smokers*	80.4 (19.1 - 338.0)	<0.001	87.1 (20.2 - 374.9)	<0.001
*Never smokers*	1		1	
Pack-years	1.04 (0.99 - 1.09)	0.058	1.06 (0.91 - 1.16)	0.067
Exhaled CO performing	1.26 (0.84 - 1.90)	0.263	0.93 (0.52 - 1.67)	0.995

The only independent factor associated with acceptance for COPD screening was the smoking status.

**Figure 2:**
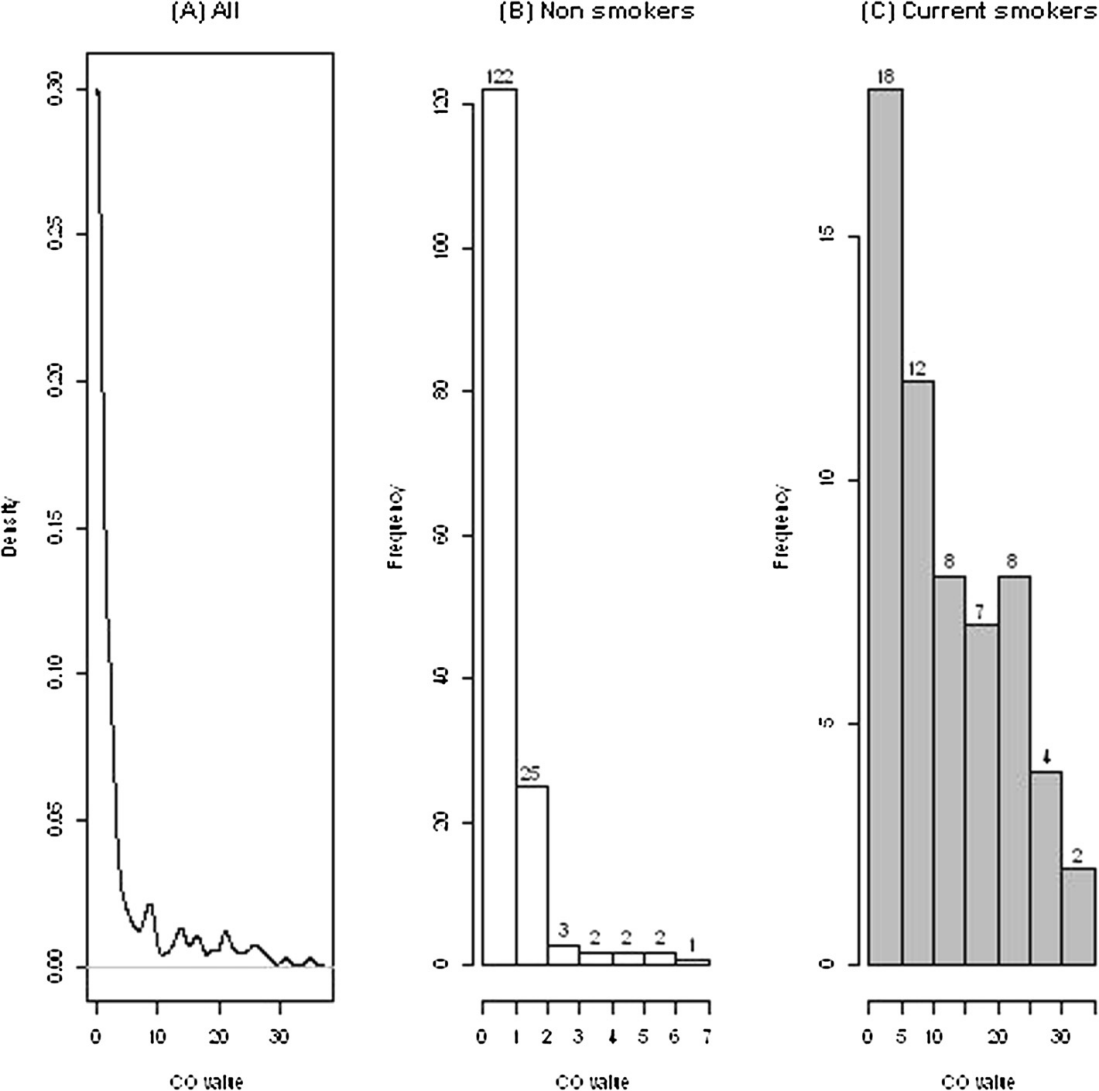
Density estimation of exhaled CO for all **(A)**, non smoker **(B)** and smoker **(C)** patients.

**Figure 3:**
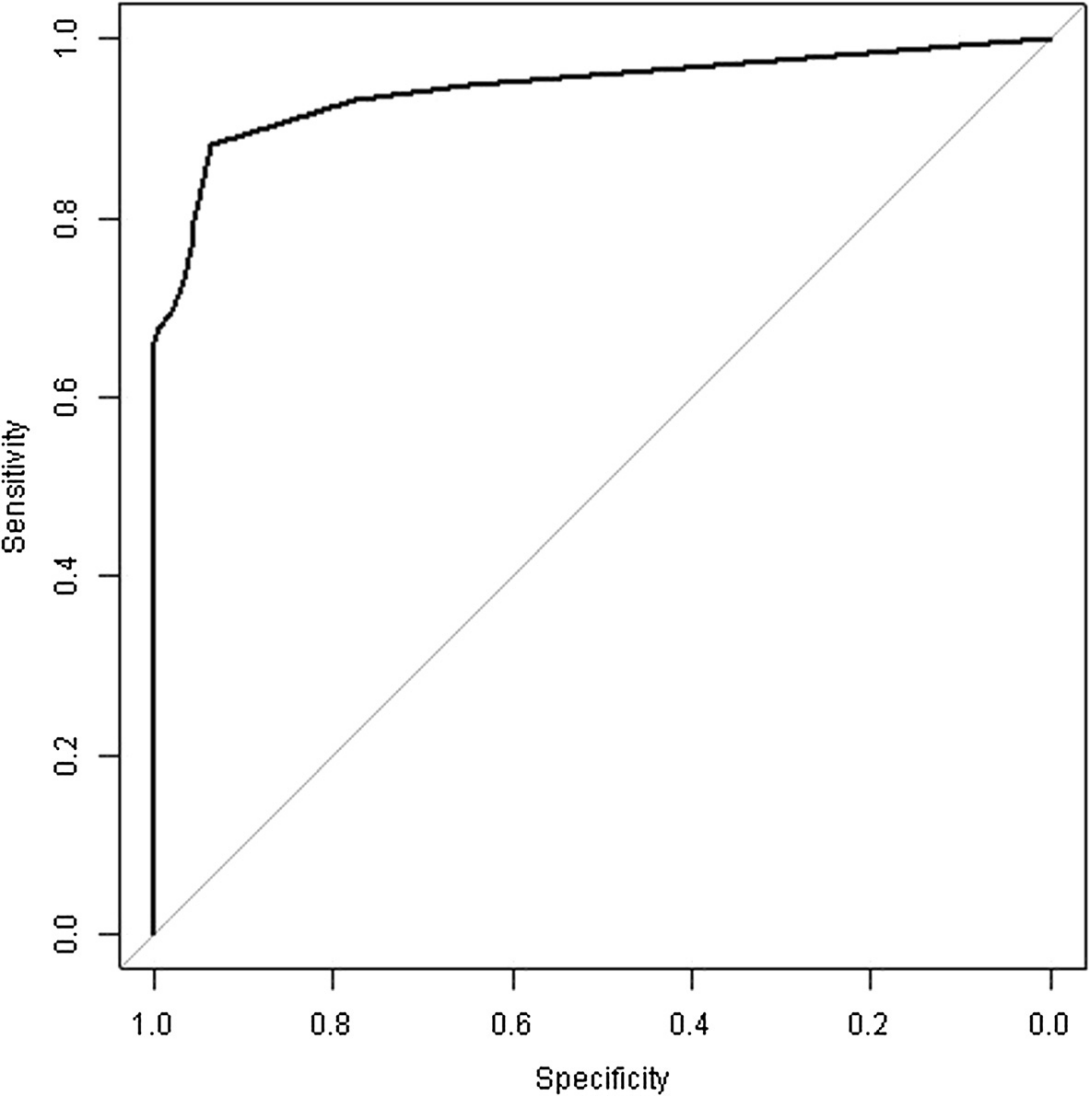
Receiver Operating Characteristic curve (ROC) exhaled CO value and current smoking status.

When assessed, mean ± standard deviation (median, interquartile range) eCO concentration was 3.88 ± 7.01 (1.00, [0 – 3.05]) exhaled CO (ppm). It reached 12.04 ± 9.00 (9.00, [4.00 – 19.00]) in current smokers, 0.82 ± 1.32 (0, [0 – 1.50]) for never smokers and 0.62 ± 1.29 (0, [0 – 1.00]) for former smokers. Figure 2 presents the distribution of the eCO for the overall population (Figure 2A), the non smokers (never and former, Figure 2B) and current smokers (Figure 2C). ROC correlating eCO value and current smoking status was performed (Figure 3), showing an optimal cutoff of eCO of over 2.5 ppm (AUC: 0.935, 95% CI [0.890-0.979], sensitivity: 0.867, specificity: 0.936, positive predictive value of 52/62 (83.9%), negative predictive value of 146/154 (94.8%)).

## Discussion

In this study, we evaluated a screening model for early detection of patients with COPD assuming that measuring eCO can lead to a debate on smoking consequences. This investigation was to address a significant correlation between eCO assessment and a debate during consultations. This outcome was not achieved even though eCO increased expression of cigarette smoking concerns. Considering that smoking remains a key contributor factor to develop COPD, it was found that CO measurement device is a significant tool inducing a large increase in smoking discussions. The measurement of exhaled CO levels is an immediate and easy method of assessing a patient’s smoking status as it may potentially introduce a climate of concern.

Our study reveals that CO measurement in medical primary care consultations (for other causes than COPD symptoms) failed to promote patients acceptance for entering into a COPD screening process, even in current/former smokers. Potentially, this observation is related to poor COPD knowledge in the general population – and a weak concern in GP’s mind, and thus no clear debate was initiated on this subject or if so, the information exchanged during the debates on COPD was shallow. Nonetheless, rates of acceptance were surprisingly high (nearly half of the smokers) meaning that both patients and GPs behaviours could have been modified by the study itself, even in the group of patients not performing eCO measurement. The main limitation of the present study therefore is related to the lack of an observation period before the study.

As a wide spread of this disease is foreseen in the future and thus an increasing demand of medical consultations is expected, these results reinforce the need to commit additional efforts among lung specialists into sharing information about COPD among general population and doctors [2].

Spirometry in primary care will probably be the next step to achieve the goal of early COPD screening. PIKO-6 and other attempts related to airflow assessment mostly failed due to absence of funding and lack of expertise even though worth results were gathered [9-16]. Shortcoming spirometric pitfalls will probably require conjugated efforts between respiratory physicians and GPs but health care networks are currently working on these aspects. Pharmacists and other health care providers are to be involved too [17-19].

The opportunity offered by the future lung cancer screening programs that will be held in western countries should not be missed. These programs will potentially promote generalized and potentially repeated CT-scans in selected populations – who share the same risk factors than for COPD (age and smoking history). Findings unrelated to lung cancer have been shown to improve health status and potentially reduce deaths also because of these unexpected findings [20-23]. Successes of these programs are based on a clear and wide awareness for cancer in the general population, largely associated with smoking. Emphysematous changes of the lung parenchyma and other findings related to smoking (airway wall thickening, suggestive signs of bronchiolitis or desquamative interstitial pneumonia for example) are potential triggers for COPD screening as this can more easily be heard and become a cause for concern for both patients and doctors than spirometry alone [22].

## Conclusions

We conclude that generalized eCO measurement in general practice failed to improve acceptance for early COPD screening. Larger studies using a comparative historical period are required to definitely conclude whether or not we should give up this very simple opportunity.
